# Supplementing Sacha Inchi (*Plukenetia volubilis*) Oil in Laying Hen Diets: Influences on Production Performance, Egg Quality and Fatty Acid Profile

**DOI:** 10.3390/vetsci12100953

**Published:** 2025-10-02

**Authors:** Nguyen Cong Oanh, Cu Thi Thien Thu, Jean-Luc Hornick, Don Viet Nguyen

**Affiliations:** 1 Faculty of Animal Science, Vietnam National University of Agriculture, Hanoi 131000, Vietnam; ncoanh@vnua.edu.vn (N.C.O.); cttthu@vnua.edu.vn (C.T.T.T.); 2 Department of Veterinary Management of Animal Resources, Faculty of Veterinary Medicine, University of Liège, 4000 Liège, Belgium; jlhornick@uliege.be; 3 School of Environmental and Rural Science, University of New England, Armidale, NSW 2351, Australia

**Keywords:** dietary inclusion, egg composition, feed conversion ratio, hybrid chicken, omega-3 polyunsaturated fatty acid, yolk

## Abstract

The omega-3 polyunsaturated fatty acids (n-3 PUFA) in animal-derived foods are increasingly important for human health. Sacha inchi (*Plukenetia volubilis* L.), rich in n-3 PUFA, is a promising feedstuff for producing n-3 PUFA-enriched animal products. However, research on dietary Sacha inchi oil (SIO) supplementation in laying hens remains limited. Therefore, this study aimed to evaluate the effects of SIO on production performance, egg quality, and yolk fatty acid (FA) composition in laying hens. A total of 192 hybrid hens [(♂ White Leghorn × ♀ Egyptian Fayoumi); initial body weight: 1910 ± 22.14 g; age: 25 weeks old] were randomly assigned one of the four diets: a basal diet (CONT), and three tested diets based on CONT with the addition of 1.5%, 3.0%, and 4.5% SIO (as-feed basis) (SI15, SI30, and SI45, respectively). Each dietary treatment included 48 hens housed in 12 battery cages for 56 days with 4 birds per cage. Increasing dietary SIO levels significantly reduced average daily feed intake (*p* < 0.05). Egg yolk weight linearly increased (*p* < 0.05) with higher SIO inclusion, while other egg quality traits were unaffected. SIO supplementation significantly increased (*p* = 0.001) yolk n-3 PUFA level (from 0.79% to 8.29% on day 28, and from 0.87% to 9.13% on day 56) and substantially reduced the n-6/n-3 PUFA ratio by about nine-fold on both 28 and 56 days. In conclusion, SIO is a promising n-3 PUFA-rich feed ingredient for egg production. Adding 3.0–4.5% SIO in laying hen diets can enhance egg yolk weight and n-3 PUFA level while reducing feed intake without negatively affecting egg production or quality.

## Simple Summary

This study examined the influences of Sacha inchi oil (SIO) inclusion in laying hen diets on their egg production, quality, and yolk fatty acid composition. A total of 192 hens were assigned to diets containing 0%, 1.5%, 3%, or 4.5% SIO over 56 days. Increasing dietary SIO levels significantly reduced feed intake while improving feed efficiency without negatively affecting egg production rates. Higher SIO inclusion led to increased yolk weight while maintaining other quality parameters. Importantly, SIO supplementation significantly enhanced yolk omega-3 polyunsaturated fatty acid (n-3 PUFA) proportion and reduced the n-6/n-3 PUFA ratio in a dose-dependent manner, contributing to healthier fatty acid profiles in eggs. The study highlights the potential of SIO as a n-3 PUFA-rich feed source to produce health benefit-added eggs. Given the increasing consumer demand for functional foods, using 3% and 4.5% SIO in laying hen diets can improve the nutritional quality of eggs by enriching them with n-3 PUFA without adverse effects on productivity. Further research is recommended to evaluate the preservation and oxidative stability of n-3 PUFA-enriched eggs produced using SIO supplementation under various storage conditions to ensure quality maintenance during shelf life.

## 1. Introduction

The stringent quality and safety standards of animal products are expected to continually improve to meet increasing consumer demands. Enriched omega-3 polyunsaturated fatty acids (n-3 PUFA) animal-derived food can provide a direct dietary source of these beneficial lipids for humans, potentially reducing the risk of various diseases [1,2,3,4]. Recent reports have showed that supplementing animal diets with n-3 PUFA-rich sources including marine algae, seafood, fish oil, flaxseed, and other oilseeds can increase the n-3 PUFA level in egg, meat, and milk [5,6,7]. The use of sea-derived products had some limitations, such as the high price of fish oils or microalgae, or undesirable fishy odour in animal products [8,9]. Consequently, researchers advocate supplementing plant-derived oils, including flaxseed oil and canola oil that are abundant in n-3 PUFA as sustainable and economic alternatives in animal diets [3,10]. In Vietnam, Sacha inchi (*Plukenetia volubilis* L.) is recognised as a highly medicinal plant and is increasingly cultivated in many areas across the country [11]. Sacha inchi seeds are rich in protein, lipids, tocopherols, flavonoids, minerals [1,12]. Moreover, Sacha inchi oil (SIO), extracted from its seeds, contains an exceptionally high PUFA content (82–93%) [10,12] and bioactive compounds such as sterols and tocopherols [13]. Particularly, SIO is predominantly rich in alpha-linolenic acid (ALA, C18:3n-3), followed by linoleic acid (LA, C18:2n-6) [11,13]. As a result, its n-6/n-3 PUFA ratio (0.81–1.12) [1] closely aligns with the optimal 1:1 ratio recommended for human health [14]. In humans, SIO consumption has been associated with reductions in blood pressure, total and low-density lipoprotein cholesterols in serum [15], as well as notable enhancements in cognitive functions, particularly memory and attention [16].

Compared with other common n-3 PUFA sources, SIO exhibits several notable advantages as a feed ingredient. Relative to flaxseed oil, SIO provides a similarly high ALA level of but offers superior oxidative stability due to its natural antioxidant content [13]. This enhanced stability helps maintain oil quality during feed storage and reduces the risk of lipid peroxidation in animal diets. In addition, SIO possesses a mild flavour and lacks the bitter taste sometimes associated with flaxseed oil, which may improve feed palatability and animal acceptance. When compared with fish oil, SIO presents a sustainable, plant-based alternative that avoids issues of marine resource depletion, potential contamination with heavy metals, and undesirable fishy odour or flavour in animal products [8,9]. These features make SIO a promising and environmentally friendly n-3 PUFA source for functional poultry diets. Many animal studies have shown that poultry fed diets supplemented with SIO present interesting results. Particularly, growth performance and feed conversion ratio (FCR) were enhanced in broilers fed a diet containing 6% SIO for 49 days compared to those fed the control diet without SIO [17]. In our recent study, replacing 2% soybean oil with SIO in combination with 1% herbal powder reduced cholesterol levels and increased n-3 PUFA content in the breast muscle of slow-growing coloured broilers [10]. Conversely, Sepúlveda et al. [18] found that including up to 9% SIO in Ross 308 broiler diets did not affect body weight gain and FCR compared with equivalent levels of palm or chicken oil. In laying hens, supplementation with 0.5% SIO enhanced consumer acceptability without introducing any undesirable off-flavours in the eggs [19]. Moreover, Zamora et al. [9] showed that hens fed a diet supplemented with SIO inclusion reduced n-6 PUFA specifically linoleic acid (LA), and increased n-3 PUFA in egg yolk. Despite these findings, most poultry studies have focused on broilers, with limited research on laying hens, particularly regarding how different dietary inclusion levels of SIO affect egg production performance, egg quality traits, and yolk fatty acid composition. Furthermore, data on the dose–response relationship between SIO supplementation and nutritional egg quality are scarce. Therefore, the aim of the present study was to assess the impacts of the dietary SIO inclusion at different inclusion levels on egg production performance, quality traits, and the fatty acid profile of laying hens.

## 2. Materials and Methods

### 2.1. Feedstuff Preparation

Sourced soybean oil (SBO) and SIO were provided by Viet Healthy Company Ltd., Hanoi, Vietnam. The oils were extracted using a cold-pressing process without additives to preserve oil quality and maintain the integrity of their fatty acid (FA) compositions. They were sampled, and their FA profiles were analysed according to the method described below. The FA profile of SBO and SIO are presented in Table 1. The total saturated fatty acid (SFA) percentage of SIO (8.83%) was markedly lower than that of SBO (33.00%), primarily because of reduced palmitic acid level (3.36% vs. 27.00%). In contrast, the total UFA of SIO (91.17%) was higher than that of SBO (67.00%), with substantially greater PUFA (77.94% vs. 32.01%). Notably, SIO was particularly rich in ALA (38.54% vs. 2.05%), resulting in a much higher n-3 PUFA content (38.54% vs. 2.07%) and a markedly lower n-6/n-3 PUFA ratio (1.02 vs. 14.46) compared with SBO. These values were consistent with recent reports [8,10,20].

Raw feed ingredients used for formulation of experimental diets were obtained from a local feed company (HanoPhavico Company Ltd., Hung Yen, Vietnam). The oils used in the experiment were kept in sealed containers and stored at room temperature (about 20–25 °C) in a well-ventilated area protected from direct sunlight to minimise oxidation.

### 2.2. Animals and Housing

The experiment was conducted from February 2024 to April 2024 at the experimental station of the Faculty of Animal Science, Vietnam National University of Agriculture, located in Gialam commune, Hanoi, Vietnam. A total of 192 hybrid laying hens (Breed: ♂ White Leghorn × ♀ Egyptian Fayoumi; age: 25 weeks old; initial body weight: 1910 ± 22.14 g) were selected from a flock that had been reared under ambient room temperatures ranging from 21 to 28 °C. The experimental hens were individually numbered using plastic leg tags. The birds were randomly assigned to four dietary treatment groups, with each group having 12 battery cages (50 cm × 50 cm × 40 cm), equipped with a feed trough (50 cm × 15 cm × 10 cm), and an automatic drinking water system, housing 4 birds per cage. Throughout the experimental period, the birds were daily exposed to 16 h of lightness and 8 h of darkness as the recommendation of Oanh and Thu [11]. Each hen was offered approximately 120 g of feed per day, split into two equal portions that were given at 07:30 h and 13:30 h throughout the experimental period. Refusals from each group were recorded daily before feeding in the morning, weighed, and kept at −20 °C until the end of the experiment for analysing dry matter. During the feeding trial, drinking water was provided ad libitum. The experiment was conducted over a 56-day period after a seven-day adaptation phase.

### 2.3. Diets

Laying hens were randomly allotted to one of four dietary treatments including a control diet (CONT) and 3 experimental diets. The CONT was composed of maize, wheat, meat and bone meal, soybean meal, and SBO. The experimental diets were based on CONT supplemented with 1.5%, 3.0%, and 4.5% SIO (as-feed basis), and they were named SI15, SI30 and SI45 diets, respectively. SIO was added to the diets daily prior to feeding.

The formulation of the CONT followed the nutrient requirements for laying hens as recommended by Leeson and Summers [21]. Raw ingredients such as maize and soybean meal were ground into a fine powder using a 2 mm screen before being blended with other feed ingredients to form a feed mixture. Then, the samples of the complete diet were collected for nutrient composition analysis. The ingredients and nutritional composition of the CONT are presented in Table 2.

### 2.4. Sampling and Measurements

#### 2.4.1. Production Parameters

Live body weight (LBW, g/bird) of experimental birds were weighed individually in the morning before feeding at the beginning (day 0) and the end of the experiment (day 56) using a Vibra DJ4000TW electronic scale (Shinko Denshi, Tokyo, Japan).

The health status of the birds in each dietary group was monitored daily and recorded using a diary agenda. The offered feed and refusals were weighed daily and recorded across diet groups to calculate the average daily feed intake (ADFI), and feed consumption for 10 eggs produced (FC, kg feed per 10 eggs) following the formulae of Phuong et al. [22]:

$$\mathrm{Feed}\;\mathrm{consumption}/10\;\mathrm{eggs}\;(\mathrm{kg})\;=\frac{Feed\;consumtion\;(kg)}{Number\;of\;eggs\;(eggs)}\times 10$$

Eggs laid were collected twice a day at 9:00 and 15:00 to calculate weekly egg production rate (%). On day 7, 14, 28, 35, 42, 49, and 56 of the experiment, 36 eggs were randomly collected from each group, and their weight was measured using a digital electronic model BL3200H scale (Shimadzu, Kyoto, Japan).

#### 2.4.2. Egg Quality

On day 28 and day 56 of the experiment, 24 eggs per diet group (2 eggs per cage × 12 cages per diet) were randomly collected to analyse egg quality parameters. Each egg was numbered and weighed using the BL3200H scale (Shimadzu, Kyoto, Japan). Egg width and length were determined by a CD-6-CSX digital electroni calliper (Mitutoyo, Kawasaki, Japan). Eggs were then broken on a glass surface to determine quality traits, following the procedure described by Lokaewmanee et al. [23]. The shape index was calculated as the ratio of egg length and width diameters. Yolk colour was assessed using the Roche yolk colour fan (Hoffmann-La Roche, Basel, Switzerland) scoring system, which includes 15 colour standards ranging from 1 (lightest) to 15 (darkest). An ORKA Digital Haugh Tester (Lakeview Dr, Bountiful, UT, USA) was employed to measure albumen height. Yolk and albumen were separated using a spoon, and the yolk was then weighed. The yolk, albumen, and shell weights (after removal of the shell membrane) were determined using the BL3200H scale (Shimadzu, Kyoto, Japan). Yolk, albumen, and shell ratios were calculated as percentages of the whole egg weight. Shell thickness was determined as the average thickness of the sharp, equatorial, and blunt regions using a digital micrometer (Mitutoyo, Kawasaki, Japan). Haugh unit (HU) was determined using the formulae of Eisen et al. [24]: HU = 100lg (h − 1.7W0.37 + 7.6), where h is the albumen height (mm), and W is the egg weight (g).

#### 2.4.3. Fatty Acid Analysis

On day 28 and 56 of the experiment, 12 eggs per diet group (1 egg per cage × 12 cage per diet) were randomly collected to determine yolk fatty acid component. Prior to analysis, two egg yolks from the same dietary group were pooled. Approximately 1 g of pooled egg yolk, and 0.2 g of SBO and SIO were solvent-extracted for total lipids using a modified Bligh and Dyer [25] method, as outlined by Nguyen et al. [26]. In brief, lipids were extracted overnight in a single-phase system using CH_2_Cl_2_:MeOH:Milli-Q H_2_O (1:2:0.8 *v*/*v*), followed by phase separation with CH_2_Cl_2_:saline Milli-Q H_2_O (1:1 *v*/*v*). The total lipids were then recovered by rotary evaporation at 40 °C. Aliquots of total lipid extracts were methylated to form fatty acid methyl esters (FAME) by heating with methanol, dichloromethane (DCM), and hydrochloric acid. FAME were then extracted into an organic solvent, washed with water, and centrifuged to separate phases. The organic layer was collected through repeated extractions, concentrated under a nitrogen gas stream. DCM, with 0.1 mL of internal injection standard (19:0 FAME) was added to grass test tubes. An Agilent 6890 plus gas chromatography system (Agilent Technologies, Palo Alto, CA, USA) equipped with a flame-ionised detector and a SP^®^-2560 capillary column (100 m × 0.25 mm i.d., 0.20 µm film thickness) was employed to analyse the FAME samples. The oven temperature was programmed at 30 °C per min from 45 °C to 140 °C (held for 5 min), then to 240 °C at a rate of 3 °C per min and held for 12 min. Helium was used as the carrier gas at a flow rate of 1.0 mL/min. The injector and detector temperatures were set at 250 °C and 270 °C, respectively. The injection volume was 1 µL in split mode (split ratio 50:1). Fatty acid proportions were calculated qualitatively using the formula: FA% = (area of individual FA/total FA area) × 100. Individually, FA that were not detected in all oils or diets, were removed from tables.

### 2.5. Statistical Analysis

The PROC mixed procedure of Statistical Analysis System (SAS) version 9.4 (SAS Institute, Cary, NC, USA) were used to statistically analysed the data on BW, feed intake, FC, egg quality parameters, and FA composition. For growth performance, each replicate cage was treated as an experimental unit, whereas individual eggs were considered experimental units for measurements of egg weight, egg quality, and FA composition. The statistical model included diet (*n* = 4) as a fixed effect, and block (*n* = 12) as a random effect. Orthogonal polynomial analyses were conducted to assess linear and quadratic responses to different dietary SIO inclusion levels. Orthogonal contrasts were used to compare data between laying hens fed diets with SIO and laying hens fed the control diet. Multiple comparisons of least-square means (LSM) were adjusted using Tukey’s method. Statistical significance was defined at *p* < 0.05, and trends were considered at 0.05 < *p* < 0.10.

## 3. Results

### 3.1. Laying Performance and Body Weight

#### 3.1.1. Bird Body Weight, Feed Intake, and Feed Consumption

The laying hens in both the control and experimental groups remained in good health throughout the experimental period, with no visible signs of diseases and mortality. No significant differences (*p* > 0.05) in LBW on day 0 and 56 between hens fed the CONT and those fed experimental diets were observed (Table 3). Initial LBW ranged from 1894 to 1931 g, and final LBW from 1942 to 1968 g.

Compared to the CONT, the ADFI decreased (linear, *p* ≤ 0.002) in laying hens fed diets with increasing inclusion levels of SIO during the first six weeks from day 1–42 of the experiment. Nevertheless, there was no significant difference in ADFI among diets during the final two weeks. Overall, a significant decrease in ADFI was observed (linear and quadratic effects, *p* ≤ 0.001) in hens fed diets with increasing levels of SIO compared to the CONT (Table 3).

There was no significant difference (linear and quadratic effects, *p* > 0.05) in FC, expressed as kg feed per 10 eggs, between hens fed experimental diets, and hens fed the CONT during any week or over the entire trial. Overall values were consistent across treatments, ranging from 1.28 to 1.30 (Table 3).

#### 3.1.2. Egg Production

Egg production rate and egg weight are presented in Table 4. The egg production rate was not significantly affected by dietary SIO inclusion at any stage of the experiment (linear and quadratic, *p* > 0.05). Over all periods, production remained high, ranging from 89.73% to 92.85%.

Across the experimental period, SIO supplementation did not linearly and quadratically affect weekly average egg weight (*p* > 0.05), despite of a slight increase with higher dietary SIO inclusion (Table 4). Overall, average egg weight increased significantly with increasing inclusion levels of SIO in the diet (Linear effect, *p* < 0.001) (Table 4). The egg weight ranged from 61.76 to 62.79 in the CONT group to 63.57–64.79 in the SI45 group from day 7 to day 56, with values showing a gradual upward trend over time in all treatments.

### 3.2. Egg Components and Quality

Dietary SIO supplementation did not significantly affect (*p* > 0.05) egg weight, shape index, yolk colour, relative albumen weight, relative shell weight, yolk ratio, albumen ratio, shell ratio, shell thickness, and Haugh units on either day 28 or day 56 (Table 5). However, relative yolk weight increased significantly with higher SIO inclusion at both time points. On day 28, hens fed the SI45 diet had linearly higher relative yolk weight compared with the control group (18.58 g vs. 17.13 g; *p* = 0.013). On day 56, this effect was more pronounced, with SI30 and SI45 groups showing significantly higher values (18.93 g and 19.41 g, respectively) than the control (17.29 g; *p* = 0.001). Overall, SIO inclusion primarily affected yolk deposition without altering other egg quality parameters.

### 3.3. Fatty Acid Profile of Egg Yolk

Fatty acid proportions of egg yolks on day 28 and day 56 are expressed in Table 6. On both 28 and 56 days, the inclusion of SIO significantly reduced (linear and quadratic, *p* < 0.001) total SFA and increased total UFA and PUFA compared with the control diet (CONT). The reduction in SFA was mainly attributable to lower palmitic acid concentrations, while the increase in PUFA was associated with higher ALA levels, leading to a marked elevation in total n-3 PUFA and a substantial decrease in the n-6/n-3 PUFA ratio. Particularly, the total n-3 PUFA proportion increased from 0.79% in the CONT to 8.29% in SI45, and the n-6/n-3 PUFA ratio decreased from 28.28 to 3.12 on day 28. The MUFA percentage, particularly oleic acid, decreased with increasing SIO supplementation levels, whereas LA increased at higher inclusion levels. Similar trends were observed on day 56, with progressive SIO inclusion lowering SFA (30.66% in the CONT vs. 27.78% in SI45) and MUFA, while increasing PUFA, ALA, and total n-3 PUFA, and reducing the n-6/n-3 PUFA ratio (26.95 vs. 2.81).

## 4. Discussion

In this study, laying hens receiving diets supplemented with SIO exhibited reduced ADFI, which is consistent with previous reports [27,28] showing that laying hens fed diets with increasing inclusion levels of different vegetables (canola or flaxseed oil) exhibited decreased ADFI during the experimental period. The reduction in ADFI with SIO supplementation may be partly attributed to the higher energy density of the diet, which allows hens to meet their energy requirements with lower intake. Additionally, dietary oils can influence satiety and gastric emptying rates and may modulate gut hormone responses such as cholecystokinin release), leading to decreased appetite. However, other findings [29,30] reported no changes in ADFI for laying hens fed diets supplemented with different fat sources (rapeseed oil, soybean, sunflower, tallow oil). Moreover, Chen et al. [31] concluded that supplementation with up to 3% SIO had no significant impact on ADFI, feed conversion rate, laying rate, or overall egg quality. Differences among the findings could be due to the various levels of dietary energy, and the percentages of SFA and UFA in the diet, which affected the digestibility and absorbability of nutrients [27,30]. Furthermore, Sepúlveda et al. [18] reported that diets containing vegetable oil sources rich in PUFA exhibited higher digestibility compared to those rich in SFA, with digestibility percentage increasing in parallel with higher n-3 PUFA levels in the diet. Similarly, Lee et al. [6] reported a decrease in ADFI with no change in body weight in hens fed flaxseed oil over 28 days. These findings align with our observations in the present study. Therefore, the improvement in nutrient digestibility and feed efficiency in response to rich n-3 PUFA oil inclusion could be attributed to lower feed consumption.

Weekly laying rate was not affected by increasing inclusion levels of dietary SIO in the present study. These results are consistent with our recent findings [11] which reported that dietary supplementation of SBO, flaxseed oil, and SIO at 1.5% did not significantly influence egg production rate. Similarly, Balevi and Coskun [32] observed no changes in egg production parameters when different oils (sunflower, cotton, corn, soybean, and tallow) were added at 2.5% of the diets. Another study conducted by Baucells et al. [29] also found no influences on egg production when laying hens were fed diets supplemented with 4% of different oils including fish oil, flaxseed oil, rapeseed oil, sunflower oil, and tallow. In contrast, relative yolk weight increased progressively with higher SIO inclusion in this study. This increase may be attributed to the elevated energy (ME) content of experimental diets, particularly due to the higher levels of unsaturated fats, which not only provide additional energy but also promote enrichment of n-3 PUFA in the yolk [33]. This observation is consistent with the finding of Grobas et al. [34], who observed that yolk weight increased in hens fed a fat-supplemented diet compared to those fed a basal diet.

Egg quality traits, such as egg weight, yolk colour and ratio, shape index, relative albumen and shell weights, albumen ratio, shell thickness and ratio, and HU, are key indicators for evaluating the impact of dietary interventions and directly or indirectly influence consumer acceptance [2,9,31]. In the present study, SIO supplementation did not affect any egg quality parameter except for relative yolk weight. Similarly, Zamora et al. [9] reported no significant effects of SIO extract supplementation on albumen weight, shell thickness, or Haugh units. Consistent with our findings, Neijat et al. [35] and Ehr et al. [36] observed no significant changes in most egg quality parameters when adding 2–3% flaxseed oil in laying hen diets. Several other studies also concluded that supplementation with n-3 PUFA-rich oils did not adversely affect egg quality [37,38,39]. The observed increase in relative yolk weight with higher dietary SIO levels in the present study may be attributed to the elevated n-3 PUFA content, particularly ALA, as Gao et al. [39] noted that most dietary ALA in laying hens is deposited directly into the egg yolk, thereby increasing yolk weight.

Variations in yolk FA composition following the addition of different oils have been previously documented [2,6,31,40]. In the present study, dietary SIO supplementation considerably improved the level of n-3 PUFA while decreasing the n-6/n-3 PUFA ratio in egg yolks as inclusion levels increased. After 28 days of the experiment, total n-3 PUFA levels in egg yolks from hens fed diets supplemented with 1.5, 3.0, and 4.5% SIO were 7.52-, 8.81-, and 10.5-fold greater, respectively, while the n-6/n-3 PUFA ratios were 7.44-, 7.56-, and 9.06-fold lower compared to the control diet. Similarly, after 56 days, n-3 PUFA levels increased by 7.59-, 8.94-, and 10.5-fold greater, and the n-6/n-3 PUFA ratios decreased by 7.74-, 7.97-, and 9.59-fold, respectively. These results align with previous findings of Chen et al. [31], who concluded that 3% SIO supplementation significantly increased yolk n-3 PUFA level while reducing the n-6/n-3 PUFA ratio. Similarly, Wang et al. [8] concluded that increasing dietary SIO levels resulted in higher total PUFA and n-3 PUFA concentrations particularly with notable increases in ALA and DHA contents and lower n-6/n-3 PUFA ratios in egg yolks. After 42 days of adding 1.5% flaxseed oil in laying hen diets, Kim et al. [41] observed an 8.53-fold increase in yolk n-3 PUFA content. The higher deposition of n-3 PUFA in egg yolks with increased dietary n-3 PUFA intake is well-established in the recent literature [2,9]. The elevated levels of n-3 PUFA detected in this study are likely due to the substantially high amount of ALA presenting in SIO. According to Gao et al. [2] and Kim et al. [41], the enrichment of PUFA in eggs from diets containing PUFA-rich plant oils may be explained by the rapid and direct absorption of ALA that avoids biohydrogenation in the gastrointestinal tract. An additional plausible mechanism is that the increase in PUFA content may also be associated with an upregulation of de novo FA synthesis [42]. Particularly, dietary inclusion of ALA-rich oils can enhance the endogenous conversion of ALA to long-chain n-3 PUFA such as EPA and DHA through hepatic elongation and desaturation pathways. The SIO inclusion may also influence the activity of lipid metabolism enzymes, including Δ5- and Δ6-desaturases, thereby promoting n-3 PUFA synthesis [43]. Furthermore, Irawan et al. [44] stated that the improved energy density and digestibility of oil-supplemented diets could enhance the bioavailability and deposition of n-3 PUFA in egg yolk. Overall, SIO supplementation effectively modified the yolk FA profile by markedly increasing n-3 PUFA proportion while lowering the n-6/n-3 PUFA ratio.

## 5. Conclusions

Dietary supplementation of SIO in laying hens effectively enhanced the nutritional quality of eggs by increasing yolk n-3 PUFA percentages and reducing the n-6/n-3 PUFA ratio, without adversely affecting LBW, egg production parameters, and overall egg quality. Increasing SIO inclusion progressively decreased ADFI and increased relative yolk weight, while leaving feed consumption per 10 eggs unchanged. The marked improvement in yolk fatty acid composition, particularly the enrichment of ALA and the substantial reduction in the n-6/n-3 PUFA ratio to values below 4/1, highlights the potential of SIO as a sustainable plant-based source of n-3 PUFA for producing value-added eggs. These findings support the inclusion of 3.0–4.5% SIO in layer diets as an effective nutritional strategy to improve the health-related lipid profile of eggs for human consumption. Further studies are required to determine the suitability of the preservation duration and oxidative stability of eggs enriched with n-3 PUFA due to SIO supplementation.

## Figures and Tables

**Table 1:** The fatty acid proportions of soybean and Sacha inchi oils, expressed as % of total fatty acids.

Fatty Acids	SBO	SIO
**Saturated fatty acids (SFA)**	33.00	8.83
Lauric acid (C12:0)	0.08	nd
Myristic acid (C14:0)	0.54	0.02
Pentadecanoic acid (C15:0)	0.03	nd
Palmitic acid (C16:0)	27.00	3.36
Margaric acid (C17:0)	0.09	0.08
Stearic acid (C18:0)	4.27	2.81
Arachidic acid (C20:0)	0.54	nd
Behenic acid (C22:0)	0.26	0.33
Lignoceric acid (C24:0)	0.12	0.19
**Unsaturated fatty acids (UFA)**	67.00	91.17
***Monounsaturated fatty acids (MUFA)***	34.99	13.24
Palmitoleic acid (C16:1)	nd	0.06
Oleic acid (C18:1n-9)	34.82	12.81
Eicosenoic acid (C20:1)	0.16	0.36
***Polyunsaturated fatty acids (PUFA)***	32.01	77.94
Linoleic acid (C18:2n-6)	29.92	39.34
Alpha linolenic acid (C18:3n-3, ALA)	2.05	38.54
Eicosadienoic acid (C20:2n-6)	0.02	0.05
Eicosapentaenoic acid (C20:5n-3, EPA)	0.01	nd
n-3 PUFA	2.07	38.54
n-6 PUFA	29.94	39.39
n-6/n-3 PUFA	14.46	1.02

nd: not detected.

**Table 2:** Ingredients and chemical composition of the basal diet.

Item	CONT
*Ingredients (as-feed basis, %)*	
Maize	52.40
Soybean meal	23.50
Wheat	10.00
Coarse limestone powder	5.00
Fine limestone powder	4.00
Soybean oil (SBO)	1.50
Meat and bone meal (MBM)	1.00
Monocalcium phosphate (MCP)	1.22
Salt (NaCl)	0.28
Vitamin Premix ^1^	0.25
Mineral Premix ^2^	0.23
Sodium Bicarbonate (NaHCO_3_)	0.26
L-Lysine	0.01
DL-Methionine	0.23
L-Threonine	0.06
Choline Chloride 60%	0.06
*Chemical composition (% DM)*	
Dry matter (DM, %)	89.5
Crude protein	16.5
Crude lipid	3.50
Total ash	14.02
Crude fibre	3.20
Ca	4.20
P	0.65
Lysine	0.90
Methionine	0.70
Methionine + Cystine	0.60
Metabolised energy (MJ/kg)	10.73

^1^ Vitamin premix in 1 kg: 15.400 IU vitamin A, 3.080 IU vitamin D3, 14 mg vitamin E, 1.4 mg vitamin K3, 1.12 mg vitamin B1, 2.8 mg vitamin B2, 3.92 mg vitamin B6, 0.014 mg vitamin B12, 56 mg niacin, 5.6 mg pantothenic acid; 0.28 mg folic acid, 0.14 mg biotin and 260.4 mg choline. ^2^ Mineral premix in 1 kg: 70 mg Mn, 50 mg Zn, 50 mg Fe, 7 mg Cu, 0.75 mg I, 0.4 mg Co and 0.17 mg Se.

**Table 3:** Effect of dietary Sacha inchi oil on live body weight, average daily feed intake and feed consumption.

Item	CONT	SI15	SI30	SI45	SEM	Linear	Quadratic
*Live body weight (LBW, g; n = 48)*							
Day 0	1931	1894	1908	1907	45.24	0.775	0.705
Day 56	1967	1968	1942	1946	46.79	0.682	0.974
*Average daily feed intake (ADFI, g/day; n = 12)*							
Day 1–7	119.7 ^a^	118.1 ^b^	118.4 ^ab^	117.4 ^b^	0.39	0.001	0.368
Day 8–14	119.4 ^a^	117.8 ^ab^	117.4 ^ab^	116.3 ^b^	0.57	0.001	0.628
Day 15–21	119.8 ^a^	117.5 ^ab^	116.5 ^bc^	114.6 ^c^	0.71	0.001	0.706
Day 22–28	119.7 ^a^	117.3 ^a^	115.6 ^b^	114.8 ^b^	0.82	0.001	0.330
Day 29–35	119.3 ^a^	116.7 ^ab^	115.1 ^b^	115.2 ^b^	0.96	0.002	0.179
Day 36–42	119.0 ^a^	116.7 ^ab^	115.3 ^b^	115.2 ^b^	0.78	0.001	0.148
Day 43–49	117.0	114.6	114.5	115.1	0.99	0.186	0.140
Day 50–56	117.2	115.5	114.7	114.7	1.21	0.127	0.464
Overall 1–56	118.9 ^a^	116.7 ^b^	115.9 ^bc^	115.4 ^c^	0.34	0.001	0.008
*Feed consumption (FC, kg feed per 10 eggs; n = 12)*							
Day 1–7	1.30	1.31	1.32	1.32	0.02	0.592	0.721
Day 8–14	1.30	1.30	1.30	1.29	0.02	0.818	0.891
Day 15–21	1.30	1.28	1.28	1.27	0.02	0.359	0.819
Day 22–28	1.29	1.29	1.27	1.27	0.02	0.258	0.900
Day 29–35	1.29	1.28	1.27	1.27	0.02	0.248	0.728
Day 36–42	1.30	1.29	1.28	1.28	0.02	0.451	0.842
Day 43–49	1.31	1.27	1.27	1.27	0.02	0.172	0.294
Day 50–56	1.30	1.28	1.28	1.27	0.02	0.316	0.694
Overall 1–56	1.30	1.29	1.28	1.28	0.01	0.224	0.569

^a,b,c^ Letters in the same row with demonstrate significant differences (*p* < 0.05); CONT: a basal diet without Sacha inchi oil (SIO) supplementation; SI15: CONT with 1.5% of added SIO; SI30: CONT with 3.0% of added SIO; CONT with 4.5% of added SIO.

**Table 4:** Egg production rate (%) and egg weight (g) affected by dietary Sacha inchi oil inclusion.

Item	CONT	SI15	SI30	SI45	SEM	Linear	Quadratic
*Egg production rate (%; n = 48)*							
Day 1–7	92.41	90.63	90.18	89.73	1.71	0.273	0.697
Day 8–14	91.96	91.07	90.63	90.19	1.52	0.398	0.884
Day 15–21	92.41	92.41	91.07	90.63	1.69	0.382	0.895
Day 22–28	92.85	91.07	91.52	90.63	1.49	0.355	0.767
Day 29–35	92.41	91.52	90.63	91.07	1.58	0.490	0.673
Day 36–42	91.96	90.63	90.18	90.18	1.66	0.437	0.687
Day 43–49	89.73	90.18	90.18	90.63	1.59	0.709	0.990
Day 50–56	90.63	90.18	89.73	90.18	1.49	0.790	0.766
*Egg weight (g; n = 36)*							
Day 7	61.80	62.24	63.27	63.57	0.58	0.123	0.900
Day 14	61.76	62.34	63.61	64.08	0.90	0.244	0.953
Day 21	62.22	63.05	63.96	64.11	0.83	0.338	0.682
Day 28	62.05	63.19	63.84	64.26	0.70	0.142	0.608
Day 35	62.48	63.71	64.13	64.26	0.79	0.371	0.493
Day 42	62.50	63.69	64.01	64.25	0.80	0.432	0.555
Day 49	62.35	63.90	64.35	64.50	0.78	0.194	0.374
Day 56	62.79	63.69	64.48	64.79	0.77	0.263	0.705

CONT: a basal diet without Sacha inchi oil (SIO) supplementation; SI15: CONT with 1.5% of added SIO; SI30: CONT with 3.0% of added SIO; CONT with 4.5% of added SIO.

**Table 5:** Effects of dietary Sacha inchi oil on egg components and quality (*n* = 24).

Item	CONT	SI15	SI30	SI45	SEM	Linear	Quadratic
*On day 28 of the experiment*							
Egg weight (g)	62.70	63.52	64.03	64.45	1.27	0.316	0.878
Shape index	1.30	1.29	1.30	1.30	0.01	0.788	0.797
Yolk colour	12.25	12.50	12.58	12.58	0.19	0.227	0.530
Relative yolk weight (g)	17.13 ^b^	17.64 ^ab^	17.99 ^ab^	18.58 ^a^	0.40	0.013	0.926
Relative albumen weight (g)	37.29	37.65	37.73	37.41	1.12	0.932	0.765
Relative shell weight (g)	8.28	8.23	8.31	8.46	0.19	0.484	0.592
Yolk ratio (%)	27.43	27.87	28.10	28.84	0.62	0.119	0.809
Albumen ratio (%)	59.33	59.12	58.89	58.02	0.80	0.253	0.683
Shell ratio (%)	13.24	13.01	13.01	13.14	0.32	0.832	0.589
Shell thickness (mm)	0.61	0.63	0.62	0.62	0.01	0.655	0.247
Haugh units	88.45	87.48	88.21	90.90	1.70	0.294	0.289
*On day 56 of the experiment*							
Egg weight (g)	62.84	63.78	64.65	65.03	1.41	0.247	0.847
Shape index	1.28	1.30	1.29	1.28	0.01	0.748	0.089
Yolk colour	12.42	12.67	12.67	12.75	0.22	0.306	0.701
Relative yolk weight (g)	17.29 ^b^	18.49 ^ab^	18.93 ^a^	19.41 ^a^	0.43	0.001	0.412
Relative albumen weight (g)	37.93	37.50	37.77	37.58	1.47	0.907	0.936
Relative shell weight (g)	7.63	7.78	7.96	8.04	0.24	0.185	0.875
Yolk ratio (%)	27.63	29.15	29.55	29.99	1.02	0.111	0.603
Albumen ratio (%)	60.20	58.64	58.09	57.64	1.16	0.123	0.641
Shell ratio (%)	12.17	12.21	12.36	12.37	0.35	0.632	0.974
Shell thickness (mm)	0.58	0.59	0.61	0.60	0.01	0.242	0.621
Haugh units	89.40	89.13	89.45	89.47	1.24	0.927	0.905

^a,b^ Letters in the same row with demonstrate significant differences (*p* < 0.05); CONT: a basal diet without Sacha inchi oil (SIO) supplementation; SI15: CONT with 1.5% of added SIO; SI30: CONT with 3.0% of added SIO; CONT with 4.5% of added SIO.

**Table 6:** Fatty acid proportions (%) of egg yolk affected by dietary Sacha inchi oil supplementation (*n* = 6).

Item	CONT	SI15	SI30	SI45	SEM	Linear	Quadratic
*On day 28 of the experiment*							
**Saturated fatty acids (SFA)**	**31.76 ^a^**	**28.30 ^b^**	**28.43 ^b^**	**28.00 ^b^**	**0.24**	**0.001**	**0.001**
Lauric acid (C12:0)	nd	0.02	0.04	0.11	0.03	na	na
Myristic acid (C14:0)	0.52 ^a^	0.11 ^b^	0.36 ^a^	0.40 ^a^	0.03	0.545	0.001
Pentadecanoic acid (C15:0)	0.06	0.05	0.04	0.05	0.01	0.271	0.076
Palmitic acid (C16:0)	24.98 ^a^	22.55 ^b^	21.78 ^bc^	21.15 ^c^	0.22	0.001	0.001
Margaric acid (C17:0)	0.16 ^ab^	0.14 ^bc^	0.13 ^c^	0.20 ^a^	0.01	0.017	0.001
Stearic acid (C18:0)	6.01 ^a^	5.41 ^b^	6.07 ^a^	6.06 ^a^	0.09	0.001	0.007
Arachidic acid (C20:0)	nd	nd	nd	0.01	0.01	na	na
Lignoceric acid (C24:0)	0.01	nd	nd	nd	0.01	na	na
**Unsaturated fatty acids (UFA)**	**68.24 ^b^**	**71.70 ^a^**	**71.57 ^a^**	**72.00 ^a^**	**0.24**	**0.001**	**0.001**
***Monounsaturated fatty acids (MUFA)***	***45.38 ^a^***	***43.53 ^b^***	***38.49 ^c^***	***37.79 ^c^***	***0.35***	***0.001***	***0.146***
Myristoleic acid (C14:1)	0.08 ^a^	0.07 ^ab^	0.06 ^ab^	0.05 ^b^	0.01	0.008	0.611
Palmitoleic acid (C16:1)	2.61 ^a^	2.69 ^a^	2.58 ^ab^	2.35 ^b^	0.06	0.007	0.027
Trans-elaidic acid (C18:1)	0.11 ^a^	0.09 ^ab^	0.08 ^ab^	0.07 ^b^	0.01	0.004	0.507
Oleic acid (C18:1n-9)	42.35 ^a^	40.49 ^b^	35.61 ^c^	35.17 ^c^	0.37	0.001	0.068
Eicosenoic acid (C20:1)	0.23 ^a^	0.18 ^ab^	0.16 ^b^	0.15 ^b^	0.01	0.001	0.224
***Polyunsaturated fatty acids (PUFA)***	***22.86 ^c^***	***28.18 ^b^***	***33.07 ^a^***	***34.21 ^a^***	***0.44***	***0.001***	***0.001***
Linoleic acid (C18:2n-6)	20.97 ^b^	21.39 ^b^	25.19 ^a^	25.08 ^a^	0.48	0.001	0.589
Alpha linolenic acid (C18:3n-3, ALA)	0.68 ^d^	5.64 ^c^	6.59 ^b^	7.98 ^d^	0.24	0.001	0.001
Beta linolenic acid (C18:3n-6)	0.12	0.09	0.10	0.10	0.01	0.144	0.055
Eicosadienoic acid (C20:2n-6)	0.17	0.13	0.15	0.17	0.01	0.495	0.008
Eicosatrienoic acid (C20:3n-6)	0.11 ^ab^	0.10 ^b^	0.12 ^ab^	0.14 ^a^	0.01	0.011	0.125
Eicosapentaenoic acid (C20:5n-3, EPA)	0.00 ^b^	0.01 ^b^	0.04 ^a^	0.04 ^a^	0.01	0.001	0.736
Docosahexaenoic acid (C22:6n-3, DHA)	0.09 ^b^	0.28 ^a^	0.30 ^a^	0.25 ^a^	0.01	0.001	0.001
n-3 PUFA	0.79 ^d^	5.94 ^c^	6.96 ^b^	8.29 ^a^	0.36	0.001	0.001
n-6 PUFA	21.87 ^b^	22.06 ^b^	25.96 ^a^	25.75 ^a^	0.49	0.001	0.693
n-6/n-3 PUFA	28.28 ^a^	3.80 ^b^	3.74 ^b^	3.12 ^b^	1.08	0.001	0.001
*On day 56 of the experiment*							
**Saturated fatty acids (SFA)**	**30.66 ^a^**	**28.14 ^b^**	**28.10 ^b^**	**27.78 ^b^**	**0.38**	**0.001**	**0.001**
Lauric acid (C12:0)	0.02	0.02	0.02	0.01	0.01	0.078	0.177
Myristic acid (C14:0)	0.46 ^a^	0.28 ^b^	0.37 ^ab^	0.38 ^ab^	0.03	0.221	0.007
Pentadecanoic acid (C15:0)	0.05	0.06	0.04	0.06	0.01	0.978	0.406
Palmitic acid (C16:0)	23.66 ^a^	22.33 ^b^	21.34 ^b^	21.03 ^c^	0.29	0.001	0.096
Margaric acid (C17:0)	0.18 ^ab^	0.15 ^ab^	0.14 ^b^	0.19 ^a^	0.01	0.043	0.008
Stearic acid (C18:0)	6.26 ^a^	5.29 ^b^	6.17 ^a^	6.11 ^a^	0.13	0.001	0.002
Arachidic acid (C20:0)	0.02	0.01	0.01	0.01	0.01	0.163	0.126
**Unsaturated fatty acids (UFA)**	**69.34 ^b^**	**71.86 ^a^**	**71.91 ^a^**	**72.22 ^a^**	**0.27**	**0.001**	**0.001**
***Monounsaturated fatty acids (MUFA)***	***45.04 ^a^***	***42.42 ^b^***	***37.68 ^c^***	***37.38 ^c^***	***0.28***	***0.001***	***0.001***
Myristoleic acid (C14:1)	0.07	0.07	0.05	0.05	0.01	0.006	0.853
Palmitoleic acid (C16:1)	2.47 ^ab^	2.63 ^a^	2.49 ^ab^	2.33 ^b^	0.06	0.092	0.035
Trans-elaidic acid (C18:1)	0.12	0.13	0.10	0.10	0.01	0.004	0.748
Oleic acid (C18:1n-9)	42.08 ^a^	39.38 ^b^	34.89 ^c^	34.74 ^c^	0.31	0.001	0.001
Eicosenoic acid (C20:1)	0.29 ^a^	0.20 ^ab^	0.16 ^b^	0.17 ^b^	0.01	0.001	0.038
***Polyunsaturated fatty acids (PUFA)***	***24.29 ^c^***	***29.44 ^b^***	***34.22 ^a^***	***34.85 ^a^***	***0.40***	***0.001***	***0.001***
Linoleic acid (C18:2n-6)	22.23 ^b^	21.92 ^b^	25.47 ^a^	24.80 ^a^	0.43	0.001	0.687
Alpha linolenic acid (C18:3n-3, ALA)	0.75 ^d^	6.19 ^c^	7.34 ^b^	8.79 ^a^	0.21	0.001	0.001
Beta linolenic acid (C18:3n-6)	0.13	0.11	0.11	0.11	0.01	0.161	0.109
Eicosadienoic acid (C20:2n-6)	0.18	0.18	0.16	0.20	0.01	0.498	0.037
Eicosatrienoic acid (C20:3n-6)	0.13	0.12	0.14	0.13	0.01	0.686	0.681
Eicosapentaenoic acid (C20:5n-3, EPA)	0.01 ^b^	0.01 ^b^	0.05 ^a^	0.05 ^a^	0.01	0.012	0.001
Docosahexaenoic acid (C22:6n-3, DHA)	0.12 ^b^	0.35 ^a^	0.39 ^a^	0.34 ^a^	0.02	0.001	0.001
n-3 PUFA	0.87 ^d^	6.61 ^c^	7.78 ^b^	9.13 ^a^	0.21	0.001	0.001
n-6 PUFA	23.22 ^b^	22.68 ^b^	26.31 ^a^	25.55 ^a^	0.41	0.001	0.801
n-6/n-3 PUFA	26.95 ^a^	3.48 ^b^	3.38 ^b^	2.81 ^b^	0.58	0.001	0.001

^a,b,c,d^ Values in the same row demonstrate significant differences (*p* < 0.05); nd: not detected; na: not available; CONT: a basal diet without Sacha inchi oil (SIO) supplementation; SI15: CONT with 1.5% of added SIO; SI30: CONT with 3.0% of added SIO; CONT with 4.5% of added SIO.

## Data Availability

The original contributions presented in this study are included in the article. Further inquiries can be directed to the corresponding author.
